# Nature and nurture: environmental influences on a genetic rat model of depression

**DOI:** 10.1038/tp.2016.28

**Published:** 2016-03-29

**Authors:** N S Mehta-Raghavan, S L Wert, C Morley, E N Graf, E E Redei

**Affiliations:** 1 The Asher Center for the Study and Treatment of Depressive Disorders, Department of Psychiatry and Behavioral Sciences, Feinberg School of Medicine, Northwestern University, Chicago, IL, USA

## Abstract

In this study, we sought to learn whether adverse events such as chronic restraint stress (CRS), or ‘nurture' in the form of environmental enrichment (EE), could modify depression-like behavior and blood biomarker transcript levels in a genetic rat model of depression. The Wistar Kyoto More Immobile (WMI) is a genetic model of depression that aided in the identification of blood transcriptomic markers, which successfully distinguished adolescent and adult subjects with major depressive disorders from their matched no-disorder controls. Here, we followed the effects of CRS and EE in adult male WMIs and their genetically similar control strain, the Wistar Kyoto Less Immobile (WLI), that does not show depression-like behavior, by measuring the levels of these transcripts in the blood and hippocampus. In WLIs, increased depression-like behavior and transcriptomic changes were present in response to CRS, but in WMIs no behavioral or additive transcriptomic changes occurred. Environmental enrichment decreased both the inherent depression-like behavior in the WMIs and the behavioral difference between WMIs and WLIs, but did not reverse basal transcript level differences between the strains. The inverse behavioral change induced by CRS and EE in the WLIs did not result in parallel inverse expression changes of the transcriptomic markers, suggesting that these behavioral responses to the environment work via separate molecular pathways. In contrast, ‘trait' transcriptomic markers with expression differences inherent and unchanging between the strains regardless of the environment suggest that in our model, environmental and genetic etiologies of depression work through independent molecular mechanisms.

## Introduction

In a 12-month period, major depressive disorder (MDD) affects 6.9% of US individuals^1^ and confers the greatest disability among any mental or behavioral disorder worldwide.^2^ The etiology of MDD is not known, but it is thought that both environmental and genetic risk factors contribute to the disease.^3, 4^ Environmental risk factors include stressful early life and aggregate lifetime stressful events, which are known to modify the molecular environment of the brain and significantly increase the likelihood of MDD onset.^5, 6, 7, 8^ The genetic contribution, or the heritability of depression, is approximated at 38%.^9^ Genetic variations underlying depression have remained elusive despite large genome-wide association studies^10, 11^ until recently, when using a very well-characterized and homogeneous patient population with severe MDD led to the identification of two candidate sequence variations significantly associated with MDD.^12^ Regarding the interaction of the genetic and environmental risk factors, mainly single gene by environment interactions have been explored;^13, 14, 15, 16^ but it is not known how the polygenic genetic risk factors interact with the environment to confer risk for MDD. Furthermore, it is not possible in human studies to answer the question whether genetically encoded depression—nature—can be aggravated by the lack of nurture, such as adverse environments, or alleviated by nurture, such as enriched environments. We are in the position to explore this quandary by placing a genetic preclinical model of MDD into either an aversive or an enhanced environment.

We use the Wistar Kyoto More Immobile (WMI) model of depression, which is derived from the Wistar Kyoto (WKY) rat strain. The WKY is an established genetic model of adult and adolescent MDD with comorbid anxiety.^17, 18, 19, 20, 21, 22, 23^ The WMI depression model was generated by a bidirectional selective breeding of the WKY based on immobility behavior in the forced-swim test (FST). Similar to MDD, WMIs respond to antidepressant treatments,^24^ show sex differences in their depression onset and in comorbid anxiety,^25^ and display dysfunctions in resting-state hippocampal connectivity.^26, 27^ The genetically similar, but behaviorally distinct WKY Less Immobile (WLIs) strain was concurrently generated and serves in these studies as the control strain for the depression-like behavior of the WMI.^24, 28^ However, the WLI retained the anxiety-like behavioral phenotype of the parental WKY strain.^25^ These WMI and WLI strains also differ in their brain and blood gene expression profiles.^28, 29^

The unique blood and brain genome-wide expression profiles of WMIs compared with WLIs were analyzed in conjunction with another study using four different strains of rats exposed to chronic restraint stress (CRS) versus nonstressed controls.^28, 29^ On the basis of the differentially expressed genes in these two studies, we developed blood transcriptomic markers for MDD, which have shown translational promise.^28, 29, 30, 31^ The blood levels of these transcripts differentiated adolescents with MDD from unaffected controls,^29^ and distinguished adults with MDD from subjects with no disorder.^30^ Thus, this panel of transcripts may serve as blood markers of MDD in humans and further validate the WMI model.

Thus, the WMIs provide a distinct opportunity to probe the consequences of gene by environment interactions. The purpose of the present study is to elucidate how adverse or enriched environments affect depression-like behavior and the level of blood biomarker transcripts in the WMI animal model and whether they exaggerate or attenuate inherent differences from its control WLIs. Environmental enrichment (EE) is proposed to be the functional opposite of stress.^32, 33^ Therefore, the evaluation of the effects of EE on these blood markers in our genetic model of depression, in conjunction with the chronic stress paradigm, will help to formulate a novel understanding of the relationship between positive and negative environments and genetic susceptibility. We hypothesize, that those transcripts whose levels change in the blood paralleling behavioral changes in either strain are potential state markers of the behavioral phenotype. We also hypothesized that those transcripts whose levels are inherently different between the strains and do not change in response to the environment are potential trait markers.

Equally important issues are how the environment affects the levels of these blood markers in the brain of the WMIs and WLIs, as well as identifying those transcripts that change in parallel in the blood and the brain. Studies have indicated that the hippocampus of patients with MDD differs anatomically, morphologically and molecularly from individuals without MDD.^34^ Hippocampal transcripts whose levels change in parallel with environment-dependent behavioral changes may either be hippocampal state markers, or causative mediators contributing directly or indirectly to the observed behavior. The transcript levels that change in parallel in the blood and the hippocampus in response to the environment, but differently in WMIs and WLIs, likely reflect their genetic differences. This study could define whether adverse or enriched environments affect the genetic animal model via the same or separate molecular mechanisms, and whether they are related to the inherent differences between the depressed WMI and control WLI as reported by the defined biomarkers.

## Materials and methods

### Animals

The Institutional Animal Care and Use Committee of Northwestern University approved all animal procedures. The male WMI and WLI rats were used for the following studies. These animals were bred from the WKY parent strain as described earlier.^24^ Briefly, the WKY inbred strain had been distributed to vendors somewhere between tenth and seventeenth generation of inbreeding. These distributed animals were known to show genetic and behavioral variability.^35, 36^ The Redei lab obtained the WKYs from Harlan Laboratories (Madison, WI, USA), where they had been additionally inbred 65 generation. However, it is not known whether the sublines Harlan obtained at the beginning of the breeding were maintained as sublines or interbred. In the Redei lab, the animals were initially generated by selective, bidirectional breeding based on FST behavior from the WKY strain. The male and female rats with the highest immobility and lowest climbing scores in the FST were mated, producing the WMI line. The male and female rats with the lowest immobility and highest climbing scores were mated, producing the WLI line. Those animals showing the most extreme FST behavior within each line were selected for breeding, specifically avoiding sibling mating until the F5 generation, when sibling mating was started.

The WMI and WLI adult male rats from the twenty-seventh to the twenty-ninth generation were exposed to daily restraint stress for 2 weeks, 2 h per day (CRS; *N*=10 per strain). The CRS animals were placed into flexible plastic bags with an opening for their mouth and nose. The animals could not turn around in this apparatus nor move the plastic bags. The restraint was conducted between 1200 and 1600 h. We used two sets of controls in this study. The first was used only as a behavioral control, and they were exposed to FST without CRS (FST-control; *N*=4 per strain). Immobility in the FST was the functional selector for the generation of the WMI and WLI strains, and they have shown consistent scores across generations. Therefore, we chose to use fewer animals from the available males of the same generation as behavioral controls as opposed to the molecular control set explained next. The control animals used for the molecular studies were behaviorally naive, no FST-controls (no FST-control; *N*=9 WLI, 10 WMI). FST was performed 2 weeks after the onset of the CRS protocol for both the CRS and FST-control groups. All the animals were killed at the same time.

Separately, the thirtieth to thirty-first generation adult male WMI and WLI rats were provided with an EE (*N*=WLI=8, WMI=15) for 1 month, and immediately following FST was conducted. The controls were housed in the standard environment and exposed to FST at the same time as the EE animals (FST-control; *N*=10 WLI, 17 WMI).

In some cases, the sample sizes were not equal between the strains because of the inherent differences in strain fecundity and therefore the number of animals born on approximately similar dates. Given that these strains are both inbred, groups were not randomized, but the animals from each litter were divided into different treatment groups. Behavioral studies had larger sample sizes than the molecular studies based on previous results.^31, 34^ For transcript level analysis, we chose samples randomly from the behavioral groups.

### Behavioral testing

The animals were tested on the FST between 0800 and 1200 h as follows. In this 2-day test, on the first day, the animals were placed into a large cylinder (30 cm × 45 cm) of 22–24 °C water for a 15-min period and then allowed to rest. Next, after 24 h, the rats were again placed into the cylinder of water for a 5-min period. The activity during the second swim test was video-recorded for subsequent scoring by a blind, trained observer. The immobility, climbing, diving and swimming behaviors were scored in 5-s bins.

### Real-time reverse transcription-polymerase chain reaction

The animals were killed by fast decapitation immediately after the FST test or for the no FST-controls immediately after removal from the homecage. The brains were immediately placed in RNALater solution (Ambion, Austin, TX, USA) and frozen at −80 °C until dissection. The trunk blood was collected into PAXgene RNA tubes (PreAnalytix, Qiagen, Hombrechtikon, Switzerland) and stored at −80 °C after 1-day incubation at room temperature. Whole hippocampi were dissected using Paxinos coordinates (dorsal hippocampus (anterior-posterior: −2.12 to −4.16, medial-lateral: 0–5.0, dorsal-ventral: 5.4–7.6) and ventral hippocampus (anterior-posterior: −4.2 to −6.0, medial-lateral: 0–5.0, dorsal-ventral: 5.4–7.6)). Hippocampal RNA was isolated using the Quick-RNA MiniPrep Kit (Zymo Research, Irvine, CA, USA). Blood RNA was isolated using the PAXgene Blood RNA Kit 50 v2 (PreAnalytix, Qiagen). Complementary DNA was synthesized as described previously.^28^ ABI 7900HT real-time cycler was used to amplify 5 ng complementary DNA using SYBR green reaction mix (ABI, Carlsbad, CA, USA). The primers were either those published previously,^28^ or were designed using the default settings in ABI's Primer Express software (version 3.0, Applied Biosystems, Foster City, CA, USA) to generate primers that amplify 80–150 bp products. The primer pairs used for each gene are listed in Supplementary Table 1.

Reverse transcription-quantitative polymerase chain reactions (RT-qPCR) were performed in triplicate and reached threshold amplification within 35 PCR cycles. The transcript levels were determined relative to 18 S (commercially available from ABI, Foster City, CA, USA) or GAPDH (IDT, as listed in Table 1) using the ΔCT method, as for each gene and each experiment, qPCR was carried out on one plate. A decrease in ΔCt indicates that relative levels of the transcript are increased, as we interpret the results using the fold change=2^−^^ΔCt^ formula. We chose the following transcriptomic biomarkers that distinguished subjects with and without MDD in the human studies:^29, 30^ Adenylate cyclase 3 (*Adcy3*), ATPase, Class VI, Type 11C (*Atp11c*), CD59 molecule, complement regulatory protein (*Cd59*), family with sequence similarity 46, member A (*Fam46a*), Cell adhesion molecule 1 (*Cadm1*), myristoylated alanine-rich protein kinase C substrate (*Marcks*), Ras association and pleckstrin homology domains 1 (*Raph1*), intracellular Toll-like receptor *7* (*Tlr7*) were all derived from the WMI genetic model of depression; and autocrine motility factor receptor, E3 ubiquitin protein ligase (*Amfr*), cerebellar degeneration-related protein 2 (*Cdr2*), cytidine monophosphate *N*-acetylneuraminic acid synthetase (*Cmas*), diacylglycerol kinase, alpha (*Dgka*), interferon regulatory factor 3 (*Irf3*)*, Kiaa1539* also known as *Fam214b* and proteasome activator subunit 1 (*Psme1*) derived from the CRS study.

### Statistical analysis

The data were analyzed using Graphpad Prism v 5.02 (GraphPad Software, La Jolla, CA, USA). All the data were analyzed by two-way analysis of variance. Bonferroni-corrected significance was reported in the figures as a result of *post hoc* analyses. When significant main effects were seen by the analysis of variance, whereas *post hoc* analysis did not show significance, hypothesis testing by two-way Student's *t*-test was carried out and reported in the figures and tables. We assumed Gaussian distribution of the data and the analysis results did not change with nonparametric testing. The variance between groups was determined in all one-way tests and was equal except as described in the Supplementary Methods. Using pre-established criteria, we excluded data that were greater than two standard deviations away from the mean, or any data clearly resulting from technical errors in the RT-qPCR assays. The statistical results are listed in detail in the Supplementary Tables 2 and 3. Ingenuity Pathway Analysis (http://www.ingenuity.com) was used to determine functional networks of the transcriptomic markers. The network *P*-values are calculated using the right-tailed Fisher's exact test and the resulting *P*-scores. P-values are derived from the Fisher's exact test: a Fisher's exact test being 21 means that the *P*-value of this specific network is e^−21^=7.58e−10. Co-expression analyses for the qPCR data were carried out for the CRS and EE experiments using Spearman correlations.

## Results

### FST behavior

**Figure 1:**
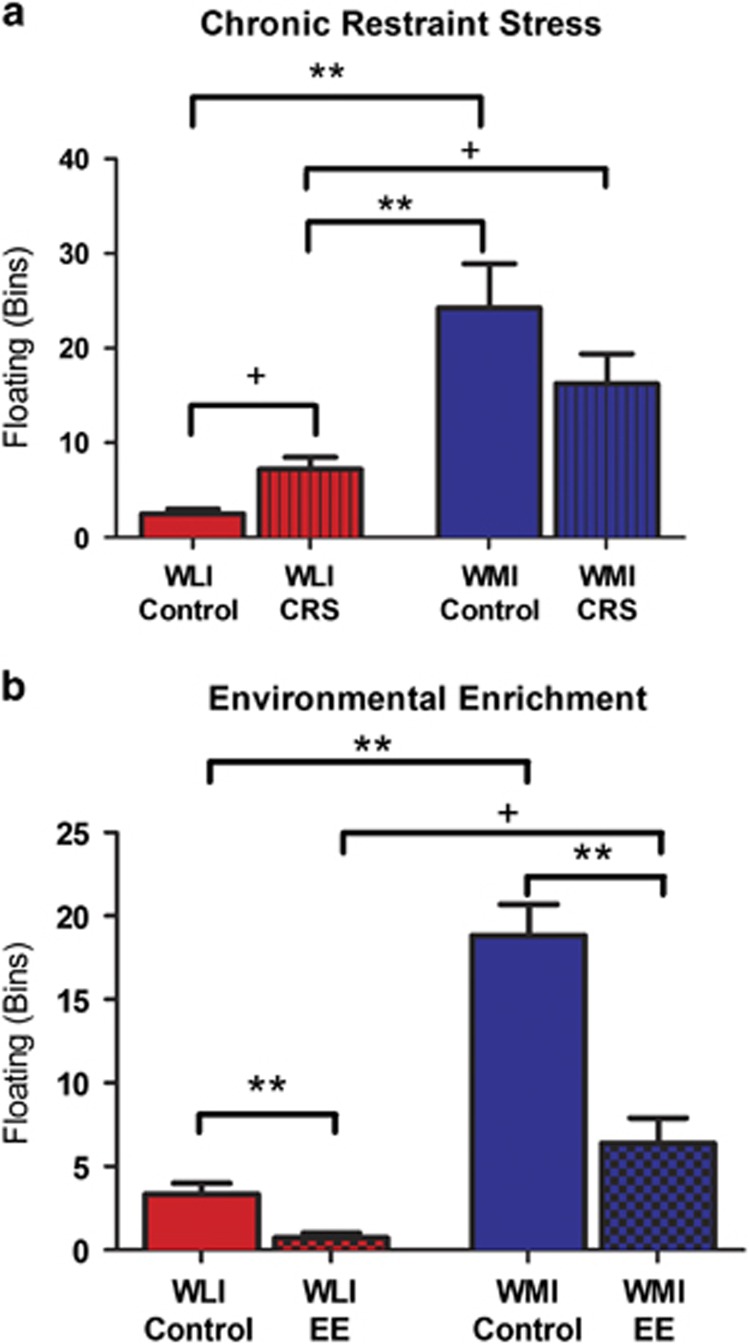
Chronic restraint stress increases immobility behavior only in WLIs while environmental enrichment reduces immobility behavior in both strains. The presence or absence of immobility behavior (floating) was registered every 5 s (bins) in the second day of the FST (**a**) immediately following the chronic restraint stress procedure. (**b**) immediately after the environmental enrichment. Data are presented as mean±s.e.m. **P<0.01 Bonferonni adjusted *post hoc*, ^+^*P*<0.05 Student's *t*-test. CRS, chronic restraint stress; FST, forced-swim test; WLI, Wistar Kyoto Less Immobile; WMI, Wistar Kyoto More Immobile.

Depression-like behavior was estimated as time spent being immobile on the second day of the FST test. In general, WMIs were significantly more immobile than WLIs (strain: CRS: F(1,21)=29.35, *P*<0.01; EE: F(1,49)=20.53, *P*<0.01; Figures 1a and b). Both CRS and EE altered immobility behavior, but in a complex interactive manner (CRS: strain × environment: F(1,21)=5.02, *P*<0.05; EE: strain × environment: *P*>0.05, NS; environment: F(1,49)=11.22, *P*<0.01). The interaction effect in the CRS study reflected the CRS-induced increased immobility of the WLIs (*t*(11)=2.44, *P*<0.05), but not the WMIs. EE decreased immobility of both strains, more apparent in the WMIs, although WLIs showed a similar effect when probed by a hypothesis testing *t*-test (Bonferroni *post hoc* WMI: *P*<0.01; WLI: *t*(17)=2.93, *P*=0.01). Neither environmental condition could override the original behavioral differences between these two genetically differing strains; immobility remained significantly different even after the CRS-induced increase or the EE-induced attenuation (Bonferroni *post hoc* CRS: *P*<0.05; Student's *t*-test EE: *t*(19)=2.53, *P*=0.02; Figures 1a and b). The CRS-induced increased immobility of the WLIs still did not reach that of WMI FST-controls (Bonferroni *post hoc* *P*<0.01; Figure 1a), but the EE-induced decrease in the WMI's immobility no longer differed from those of WLI FST-controls (Figure 1b).

### Expression of biomarker transcripts in the blood

In the CRS study, we made the assumption that the acute stress of FST following 2 weeks of CRS would not significantly affect the chronic stress state of the animals. Therefore, for the transcript analysis, the comparison was made between the CRS+FST animals and the no FST-controls.

Strain differences between the no FST-controls were found in the blood transcript levels of *Adcy3, Atp11c, Cd59, Cdr2* and *Fam46a* (Supplementary Table 4), while overall strain differences were seen in the transcript levels of *Cd59*, *Irf3, Raph1* and *Tlr7* (Table 1 and statistics in Supplementary Table 2). Chronic restraint stress significantly altered overall expression of *Cmas* and *Raph1* (Supplementary Table 2), but strain-specific CRS effects were also observed and analyzed by strain by environment interactions or by hypothesis testing (Supplementary Table 2). Specifically, CRS increased the expression of *Cmas, Fam46a* and *Tlr7* in the WLIs, without changing them in the WMIs. Conversely, blood levels of both *Adcy3* and *Raph1* increased in the WMIs but not the WLIs.

In the EE experiment, overall strain differences were seen in the blood transcript levels of *Amfr, Cmas, Cadm1* (Table 2 and Supplementary Table 3), differing from the strain differences of *Adcy3, Cd59* and *Fam46a* in the FST-controls (Supplementary Table 4). Environmental enrichment increased blood transcript levels of *Raph1* in both strains (Table 2 and Supplementary Table 3). However, EE affected levels of *Amfr*, *Cdr2*, *Cadm1*, *Irf3*, *Marcks* and *Tlr7* in a strain-dependent manner (Supplementary Table 3). Specifically, EE induced decreases in transcript levels of *Amfr*, *Cdr2* and *Marcks*, but increases in levels of *Cadm1* and *Irf3* in WLIs only. In contrast, *Cadm1* was decreased while levels of *Tlr7* were increased in response to EE in WMIs only. EE did not reverse control strain differences in transcript levels of these blood markers.

The comparison of gene expression in the blood of the no FST- and the FST-controls in the CRS and the EE studies, respectively, highlighted that *Adcy3*, *Cd59* and *Fam46a* transcript level differences between WLIs and WLIs were inherent and independent of the acute stress of FST, whereas levels of *Atp11c* and *Cdr2* were responsive to FST (Supplementary Table 4).

### Expression of biomarker transcripts in the hippocampus

In the CRS study, strain differences in hippocampal expression of *Cd59*, *Cdr2*, *Cadm1, Irf3*, *Marcks* and *Raph1* were present in the no FST-controls (Supplementary Table 5), and after CRS, overall strain effects were maintained in *Cd59, Cdr2, Irf3 and Raph1* (Table 3 and Supplementary Table 2). Additional overall strain effects were also found for *Atp11c, Cmas, Dgka* and *Psme1* (Table 3 and Supplementary Table 2). In response to CRS, *Amfr* and *Raph1* expression increased, and levels of *Atp11c* decreased in both strains (Table 3). CRS affected hippocampal levels of *Cmas*, *Kiaa1539*, *Fam46a*, *Cadm1* and *Irf3* in a strain-dependent manner. Specifically, transcript levels of *Cmas* and *Kiaa1539* increased only in the WLIs, while levels of *Fam46a* and *Irf3* decreased and *Cadm1* increased only in WMIs in response to CRS. *Marcks* expression decreased in the WLI but increased in the WMI CRS hippocampi.

Importantly, control strain differences in the expression of *Cd59* and *Cdr2* along with the CRS-induced increased expression of *Cmas* and *Raph1* in WLIs and *Raph1* in WMIs were common between hippocampus and blood.

In the EE experiment, of the FST-control strain differences in transcript levels of *Dgka*, *Fam46a, Irf3, Kiaa1539* and *Marcks* (Supplementary Table 5), overall strain effects for *Dgka, Fam46a* and *Irf3* were maintained (Table 4 and Supplementary Table 3). Environmental enrichment affected hippocampal transcript levels of *Adcy3*, *Cdr2*, *Dgka*, *Cadm1*, *Irf3*, *Kiaa1539*, *Psme1, Raph1* *and Tlr7* (Table 4 and Supplementary Table 3). In response to EE, levels of *Adcy3* and *Kiaa1539* decreased in both WLI and WMI hippocampi. EE affected the expression of *Dgka*, *Cadm1*, *Psme1, Raph1* and *Tlr7* differently between the strains (Supplementary Table 3). Specifically, the EE animals showed increased levels of *Dgka, Irf3*, *Psme1* and *Raph1* and decreased levels of *Tlr7* in WLIs only, while EE decreased levels of *Cadm1* in WMIs only.

EE-induced increases in levels of *Raph1* and *Irf3* in the WLI, and decreases in *Cadm1* in the WMIs were common to hippocampus and blood.

The comparison of gene expression in the hippocampus of the no FST- and the FST-controls in the CRS and the EE studies, respectively, highlighted that *Irf3* and *Marcks* transcript level differences between WLIs and WLIs were inherent and independent of the acute stress of FST, while hippocampal levels *Cadm1*, *Cd59*, *Cdr2*, *Dgka*, *Kiaa1539*, *Fam46a*, *Raph1* were responsive to FST (Supplementary Table 5).

### Pathway analysis of blood transcripts

**Figure 2:**
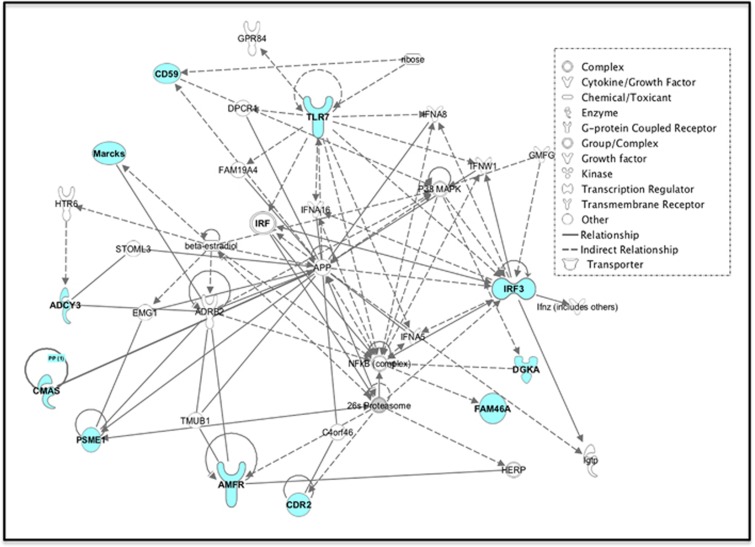
Network analysis of blood transcripts. Interacting networks generated using Ingenuity Pathway Analysis were enriched for functions: gene expression, cell-to-cell signaling and interaction, hematological system development and function. The right-tailed Fisher's exact test of this network is 21, equivalent to *P*=7.58e−10. Inset legend shows symbol designations. ADRB2, beta-2 adrenergic receptor; APP, amyloid precursor protein; IRF3, interferon regulatory factor 3; MAPK, mitogen-activated protein kinase; NF-κB, nuclear factor kappa B.

All the transcripts were entered into Ingenuity Pathway Analysis. Figure 2 displays the most significant interacting network. Highlighted is the interconnectivity of the markers through mitogen-activated protein kinase, beta-2 adrenergic receptor, amyloid precursor protein, nuclear factor kappa B, as well as *Irf3* and *Tlr7*, two of the measured markers. Further, five of the markers, *Cd59, Cdr2, Irf3, Dgka* and *Fam46a,* which retained their basal hippocampal differences even after environmental manipulation, are members of this network.

### Co-expression network

The frequency distribution of correlation coefficients of blood transcripts were presumed to differ only between the WLI no FST-control and the WLI CRS animals as CRS induced a behavioral change in WLIs. However, no change was observed in the architecture of the co-expression network by CRS in WLIs (Supplementary Figure 1a). In contrast, after EE, the frequency distribution of correlation coefficients significantly shifted to greater *r* in both WMIs (*t*(180)=3.66, *P*<0.001) and WLIs (*t*(208)=3.83, *P*<0.001; Supplementary Figure 1b) in parallel to the behavioral change, indicating greater interconnections between the transcripts.

Co-expression analyses of blood transcript levels resulted in unique expression networks for WLIs following CRS, which did not overlap with those of the WLI no FST-controls, or WMIs (Supplementary Figure 2a). Notably, expression of all three, *Cmas*, *Tlr7* and *Fam46a,* WLI-specific transcripts that changed significantly by CRS correlated with each other via *Atp11c*. Unique expression networks were also found in WMI blood following EE (Supplementary Figure 2b) that did not overlap with those of WMI FST-controls or WLIs. This correlation network connected *Cadm1* and *Tlr7*, transcripts with significant changes after EE in the WMIs, emphasizing them as ‘hubs'.

## Discussion

In the current study, we identified that chronic restraint stress-induced elevation in depression-like behavior is strain dependent, while environmental enrichment-mediated attenuation of this behavior is independent of genetic background. CRS-induced behavioral changes did not compound genetic susceptibility to depression in the WMI model. Consistent with the lack of an additive effect in the behavior of the WMIs and the increased depression-like behavior of the WLIs, expression differences between WMIs and WLIs in depression blood biomarkers were not exaggerated by CRS. Similarly, parallel to the EE-mediated decrease in depression-like behavior in both WMIs and WLIs, blood transcript level differences between the strains were not attenuated. The opposing behavioral outcomes of CRS and EE in the WLIs are not reflected in inverse changes of blood or hippocampal gene expression profiles. These findings advance our understanding by suggesting that CRS and EE likely generate their behavioral outcomes in this model via diverse molecular mechanisms, and these are inherently different from the transcriptomic manifestation of genetic differences between the depressed WMI and control WLI.

Strain-specific responses to the environment occur in the depression-like behavior of phylogenetically separate strains. Specifically, chronic stress has been shown to elicit strain-dependent changes in immobility behavior.^37, 38^ In genetic models of depression, chronic mild stress increases anhedonic behavior of the Flinders sensitive and the WKY genetic rat models of depression,^39, 40^ but has no effect on the Swim Low-active rats.^41^ EE has been shown to attenuate depression-like behaviors in Sprague Dawley rats,^42, 43, 44, 45^ and ameliorate learned helplessness in rats bred for high learned helplessness behavior.^46^ Should a genetic model of depression with high stress-reactivity, including the WMIs, be exposed to CRS, CRS would not likely have any further effect on their depression-like behavior. In agreement with this scenario, and the inoculation stress hypothesis of environmental enrichment,^47^ WMIs only showed alteration of depression-like behavior following EE. Increased neurogenesis is one of the proposed mechanisms by which EE attenuates depression-like behavior.^48, 49^ This mechanism implicates the hippocampus, which is known to be affected in MDD, and is known to be particularly sensitive to EE.^50^ Still undetermined is whether hippocampus-dependent enhancement of learning and memory, characteristic consequences of EE,^51, 52^ are the direct cause of attenuated depression-like behavior.

Our initial studies aiming to identify a candidate blood biomarker panel are based on the unbiased, exploratory exploitation of the genetic and environmental components of MDD etiology by two different animal models. The results of these studies present two sets of candidate blood biomarkers whose expression differences do not overlap.^28, 29^ This provides the initial evidence that the molecular signatures of CRS-induced and genetically conferred depression-like behavior differ. Nevertheless, it is surprising that in the current study, the CRS-induced changes in blood transcript levels were neither additive nor synergistic to the inherent biomarker signature of WMIs. However, blood transcript levels of *Fam46a* and hippocampal expression of *Marcks* mirrored the CRS-induced behavioral change of WLIs, as WLI levels of these transcripts approached those of WMIs, suggesting these as state markers in the blood and the hippocampus, respectively.

On the basis of the opposite behavioral changes induced by CRS and EE in the WLIs, we expected to find transcript level changes in response to EE in the WLIs to be opposite to those in response to CRS, and those in response to EE in the WMIs to approximate the levels of WLI controls. Although we found no convincing evidence to support this original hypothesis, the parallel decreases in FST immobility in both WMIs and WLIs after EE were mirrored by increases in blood transcript levels of *Raph1* and hippocampal levels of *Adcy3* and *Kiaa1539* in both strains. These findings implicate *Raph1, and Adcy3* and *Kiaa1539* as state markers in the blood and in the hippocampus, respectively.

The next significant question was the overlap between blood and hippocampal expression of markers and their responses to the environment. Here we showed that both blood and hippocampal levels of *Cd59*, *Cdr2* and *Fam46a* differed between control WMIs and WLIs. Furthermore, transcript levels of *Cmas*, *Cadm1*, *Irf3* and *Raph1* changed in the same direction in response to the environment in both tissues, but differently in WMIs and WLIs. The expression differences of these latter transcripts in both the blood and the hippocampus might be a direct or indirect consequence of the genetic differences between the WMI depression model and its control inbred strain.

It is of interest that all of these genes have confirmed or suggested roles in neurodevelopment and neurodegeneration. The enzyme that *Cmas* (cytidine monophosphate *N*-acetylneuraminic acid synthetase) encodes regulates brain sialylation levels and, therefore, affects brain development and neurodegeneration.^53^ In the central nervous system, *Cadm1* is involved in neural cell adhesion processes and synaptogenesis,^54^ is thought to contribute to autism spectrum disorder,^55^ and has also been linked with social impairments and anxiety-like behavior.^56^ *Irf3* activates the transcription of several interferon-induced genes and it has been implicated in brain function and dysfunction such as hippocampal network excitability.^57^ *Irf3* has also been shown to have expression association with schizophrenia.^58^ *Raph1* is also known as amyotrophic lateral sclerosis 2 (juvenile) chromosome region, candidate 9. Hippocampal expression of *Raph1* is thought to be involved in suicide-related processes independent of depressive psychopathology.^59^ Interestingly, *Raph1* has also been associated with metabolic activity^60^ and involved in neuronal migration.^61^ These genes are exciting targets for future study and are excellent candidates for potential therapeutic intervention.

Although pathway analysis is usually conducted with genome-wide expression data, using the limited number of biomarker candidates here proved to be thought provoking. Trait markers can be defined as those transcripts that retained their basal expression differences between WMIs and WLIs regardless of adverse or protective environment-induced changes. These included *Cd59* in the blood and *Cd59, Cdr2, Irf3, Raph1, Dgka* and *Fam46a* in the hippocampus. All of these transcripts, with the exception of *Raph1*, showed direct or indirect relationships with amyloid precursor protein. Mutations in amyloid precursor protein have been associated with several neurodegenerative diseases,^62^ and amyloid precursor protein has also been implicated in the consequences of chronic stress.^63, 64^

In the unique co-expression network of the WLI CRS group, *Atp11c* transcript levels changed in tandem with three other significantly altered transcripts, suggesting that stress-induced depression-like behavior, as shown by the WLIs, can be ‘marked' separately from the genetic model. In contrast, in the co-expression network of the WMI EE, *Tlr7* and *Cadm1* co-expressed with several other genes, representing a signature of gene by protective environment interaction in this genetic model.

This study has several limitations, which as always, encourages future investigations. First, as only the FST test was used, which was the basis of the original selective breeding,^24^ future studies could test the effect of adverse or protective environments on other behaviors including those commonly comorbid with depression such as anxiety and memory impairment. In addition, although hippocampal abnormalities have been validated in depressed populations, there are other brain regions including the amygdala and dorsolateral prefrontal cortex, which have been shown to be associated with depression, and therefore should be explored.

Strain differences in some of the biomarker levels diverged from previous studies.^28, 29^ These differences may be due to the well-known lability of ΔCT measurements by RT-qPCR, which could be overcome in the future by the use of absolute quantification or digital PCR. As we do not know the prevalence of specific isoforms in blood or hippocampus, discrepancies between studies can result when using different primers probing different regions of the transcript. Therefore, we would first need to discover splice variants and isoforms for all of these rat transcripts before we can identify tissue-specific expression of isoforms and their regulation. The significance of this is illustrated when only one isoform exists for both human and rat transcripts, such as in the case of *Fam46a,* the strain differences between WMI and WLI are the same regardless of which part of the mRNA is amplified. However, when multiple human transcripts are described, which are not mirrored by the much lower sequencing coverage of the rat genome, differences arose using alternative primers. Exploring the isoforms of these depression markers and their regulation in the rat brain and blood followed by in the human brain and blood in health and disease will further the specificity and precision of their future diagnostic potential for MDD.

The present study exploited a genetic animal model of depression and its genetically close control to address the power of environmental nurture over a genetic predisposition. The results suggest that the two etiologies currently accepted for depression, environmental and genetic, likely work through independent molecular mechanisms that may interact. Environmental enhancements can be protective as has been suggested by human studies in which the effects of adverse early environments are reversed later by nurturing environments. Our converging evidence signifies that CRS and EE do not add to or reverse, respectively, the genetic contribution-induced behavioral and transcriptional characteristics of our animal model of disease. CRS and EE do not result in opposing changes in blood transcript levels. Depression is a very heterogeneous disease, and our blood-based biomarkers differentiated depressed and nondepressed subjects in a genetically very heterogeneous population. Here, in this genetically homogenous animal study, we have shown that environment can affect levels of these biomarkers in the periphery and the brain. The environment, therefore, can alter genetic predisposition, as the classic nature vs nurture debate has always been resolved. As environment is always at work, the gene × environment interaction is a moving target. Nevertheless, current epigenetic techniques could identify areas in the genome that are most responsive to the environment in individuals with genetic predisposition to depression and in those without it.

## Figures and Tables

**Table 1:** Blood transcript levels in WLIs and WMIs in response to CRS

*Gene*	*WLI ΔCt±s.e.m.*		*WMI ΔCt±s.e.m.*	
	*Control*	*CRS*	*Control*	*CRS*
*Adcy3*	13.13±0.24	13.44±0.51	14.10±0.28	12.56±0.38*
*Amfr*	2.35±0.17	2.64±0.24	2.40±0.15	2.67±0.17
*Atp11c*	7.72±0.10	7.23±0.37	7.04±0.14	7.40±0.53
*Cadm1*	6.99±0.11	7.09±0.29	6.76±0.14	6.80±0.17
*Cd59*	8.35±0.25	8.26±0.38	9.42±0.35	8.74±0.39
*Cdr2*	0.96±0.15	1.44±0.26	1.38±0.13	1.33±0.08
*Cmas*	5.22±0.16	4.50±0.25*	4.90±0.20	4.58±0.12
*Dgka*	2.39±0.10	2.63±0.21	2.20±0.16	2.22±0.24
*Fam46a*	5.00±0.12	4.50±0.17*	4.45±0.16	4.59±0.09
*Irf3*	3.10±0.16	3.51±0.20	2.82±0.17	2.54±0.25
*Kiaa1539*	3.01±0.18	3.11±0.19	2.86±0.07	3.15±0.17
*Marcks*	4.36±0.19	4.35±0.20	5.06±0.54	4.24±0.19
*Psme1*	3.66±0.05	3.72±0.10	3.66±0.21	3.29±0.19
*Raph1*	6.33±0.10	6.41±0.15	6.31±0.06	5.71±0.11**
*Tlr7*	5.20±0.10	4.67±0.10^++^	5.17±0.14	5.44±0.32

Abbreviations: CRS, chronic restraint stress; WLI, Wistar Kyoto Less Immobile; WMI, Wistar Kyoto More Immobile.

Please note that the ΔCt values relate to relative concentration by 2^−ΔCT^.

*P<0.05, **P<0.01 by Bonferroni *post hoc* after significant two-way analysis of variance (strain × environment). Comparisons are within strain and between control and CRS.

^++^*P*<0.01 by Student's *t*-test. Comparisons are within strain and between control and CRS.

**Table 2:** Blood transcript levels in WLI and WMIs in response to EE

*Gene*	*WLI ΔCt±s.e.m.*		*WMI ΔCt±s.e.m.*	
	*Control*	*EE*	*Control*	*EE*
*Adcy3*	11.43±0.05	11.52±0.08	11.68±0.05	11.54±0.11
*Amfr*	4.38±0.12	5.72±0.23**	4.21±0.22	4.93±0.24
*Atp11c*	7.39±0.10	7.21±0.07	7.33±0.07	7.25±0.05
*Cadm1*	5.93±0.08	5.60±0.05*	5.82±0.09	6.13±0.07*
*Cd59*	6.31±0.07	6.84±0.41	6.60±0.09	6.72±0.18
*Cdr2*	3.75±0.11	4.43±0.17**	3.81±0.10	3.90±0.19
*Cmas*	7.65±0.10	7.75±0.20	7.45±0.05	7.15±0.30
*Dgka*	4.90±0.09	4.73±0.25	4.74±0.11	4.49±0.03
*Fam46a*	6.78±0.08	6.84±0.15	7.02±0.08	6.95±0.06
*Irf3*	5.73±0.15	4.56±0.35**	5.25±0.21	4.88±0.14
*Kiaa1539*	4.83±0.12	5.22±0.18	4.93±0.16	4.77±0.10
*Marcks*	6.13±0.12	7.57±0.29**	6.45±0.32	7.06±0.26
*Psme1*	5.48±0.09	5.23±0.18	5.54±0.09	5.23±0.27
*Raph1*	10.00±0.09	9.47±0.14*	10.05±0.16	9.45±0.16*
*Tlr7*	6.81±0.11	6.93±0.11	7.23±0.31	6.24±0.34*

Abbreviations: EE, environmental enrichment; WLI, Wistar Kyoto Less Immobile; WMI, Wistar Kyoto More Immobile.

Please note that the ΔCt values relate to relative concentration by 2^−ΔCT^.

*P<0.05, **P<0.01 by Bonferroni *post hoc* after significant two-way analysis of variance (strain × environment). Comparisons are within strain and between control and EE.

**Table 3:** Hippocampal transcript levels in WLIs and WMIs in response to CRS

*Gene*	*WLI ΔCt±s.e.m.*		*WMI ΔCt±s.e.m.*	
	*Control*	*CRS*	*Control*	*CRS*
*Adcy3*	7.54±0.11	7.32±0.07	7.29±0.05	7.47±0.11
*Amfr*	5.37±0.12	4.71±0.16^++^	6.00±0.41	4.78±0.26**
*Atp11c*	6.82±0.11	7.51± 0.09*	6.98±0.10	8.40±0.27**
*Cadm1*	3.93±0.04	3.98±0.11	4.32±0.11	3.69±0.15**
*Cd59*	5.90±0.40	5.55±0.25	8.02±0.32	8.53±0.43
*Cdr2*	6.95±0.29	7.75±0.33	8.64±0.32	8.08± 0.36
*Cmas*	5.78±0.10	5.39±0.16*	5.93±0.08	6.02±0.11
*Dgka*	11.21±0.12	11.33±0.25	11.66±0.23	11.73±0.17
*Fam46a*	12.87±0.44	14.06±0.29	12.68±0.28	13.68±0.34^+^
*Irf3*	4.26±0.11	5.05±0.45	5.45±0.45	7.04±0.38*
*Kiaa1539*	7.99±0.03	7.61±0.15^+^	8.19±0.08	7.66±0.34
*Marcks*	5.31±0.09	6.02±0.19*	5.85±0.09	4.95±0.27**
*Psme1*	6.05±0.10	5.78±0.11	6.20± 0.08	6.31±0.20
*Raph1*	7.50±0.17	6.61±0.14*	9.37±0.19	8.65±0.38*
*Tlr7*	9.97±0.17	10.15±0.47	10.34±0.41	10.39±0.43

Abbreviations: CRS, chronic restraint stress; WLI, Wistar Kyoto Less Immobile; WMI, Wistar Kyoto More Immobile.

Please note that the ΔCt values relate to relative concentration by 2^−ΔCT^.

*P<0.05, **P<0.01 by Bonferroni *post hoc* after significant two-way analysis of variance (strain × environment). Comparisons are within strain and between control and CRS.

^+^*P*<0.05, ^++^*P*<0.01 by Student's *t*-test. Comparisons are within strain and between control and CRS.

**Table 4:** Hippocampal transcript levels in WLIs and WMIs in response to EE

*Gene*	*WLI ΔCt±s.e.m.*		*WMI ΔCt±s.e.m.*	
	*Control*	*EE*	*Control*	*EE*
*Adcy3*	8.21±0.05	8.54±0.08**	8.26±0.04	8.54±0.06**
*Amfr*	3.13±0.06	3.18±0.07	3.27±0.05	3.27±0.09
*Atp11c*	7.83±0.15	8.04±0.22	7.74±0.14	7.69±0.13
*Cd59*	5.39±0.08	5.44±0.10	5.49±0.06	5.41±0.08
*Cadm1*	3.13±0.06	3.28±0.08	3.17±0.10	3.45±0.07*
*Cdr2*	9.50±0.20	8.83±0.24	9.25±0.11	8.82±0.19
*Cmas*	6.38±0.11	6.36±0.19	6.30±0.11	6.47±0.14
*Dgka*	7.25±0.09	6.72±0.09**	6.85±0.10	6.69±0.02
*Fam46a*	10.63±0.12	10.50±0.18	10.07±0.14	10.29±0.17
*Irf3*	8.66±0.17	7.93±0.17*	7.92±0.18	7.55±0.17
*Kiaa1539*	7.20±0.07	6.67±0.10**	6.98±0.07	6.64±0.09**
*Marcks*	4.76±0.06	4.90±0.08	5.02±0.04	4.92±0.13
*Psme1*	7.49±0.12	7.13±0.10^+^	7.34±0.11	7.05±0.11
*Raph1*	5.48±0.06	5.00±0.08**	5.34±0.10	5.24±0.10
*Tlr7*	9.63±0.14	10.44±0.17*	9.97±0.22	10.05±0.17

Abbreviations: EE, environmental enrichment; WLI, Wistar Kyoto Less Immobile; WMI, Wistar Kyoto More Immobile.

Please note that the ΔCt values relate to relative concentration by 2^−ΔCT^.

*P<0.05, **P<0.01 by Bonferroni *post hoc* after significant two-way analysis of variance (strain × environment). Comparisons are within strain and between control and EE.

^+^*P*<0.05 by Student's *t*-test. Comparisons are within strain and between control and EE.

## References

[bib1] Substance Abuse and Mental Health Services Administration. Results from the 2013 National Survey on Drug Use and Health: Mental Health Findings. 2014. [cited 25 May 2015] Available at http://www.samhsa.gov/data/sites/default/files/NSDUHmhfr2013/NSDUHmhfr2013.htm. 27656739

[bib2] World Health Organization. The Global Burden of Disease: 2004 update. 2004. [cited 15 April 2015] Available at http://www.who.int/healthinfo/global_burden_disease/2004_report_update/en/.

[bib3] Saveanu RV, Nemeroff CB. Etiology of depression: genetic and environmental factors. Psychiatr Clin North Am 2012; 35: 51–71. 22370490 10.1016/j.psc.2011.12.001

[bib4] Heim C, Binder EB. Current research trends in early life stress and depression: review of human studies on sensitive periods, gene-environment interactions, and epigenetics. Exp Neurol 2012; 233: 102–111. 22101006 10.1016/j.expneurol.2011.10.032

[bib5] Sasagawa Y, Akai T, Nakada S, Minato H, Tachibana O, Nojima T et al. Narrow band imaging-guided endoscopic biopsy for intraventricular and paraventricular brain tumors: clinical experience with 14 cases. Acta Neurochir (Wien) 2014; 156: 681–687. 24445733 10.1007/s00701-014-1995-y

[bib6] Kupfer DJ, Frank E, Perel JM. The advantage of early treatment intervention in recurrent depression. Arch Gen Psychiatry 1989; 46: 771–775. 2774846 10.1001/archpsyc.1989.01810090013002

[bib7] Hammen C. Stress and depression. Ann Rev Clin Psychol 2005; 1: 293–319. 17716090 10.1146/annurev.clinpsy.1.102803.143938

[bib8] de Kloet ER, Joels M, Holsboer F. Stress and the brain: from adaptation to disease. Nat Rev Neurosci 2005; 6: 463–475. 15891777 10.1038/nrn1683

[bib9] Kendler KS, Gatz M, Gardner CO, Pedersen NL. A Swedish national twin study of lifetime major depression. Am J Psychiatry 2006; 163: 109–114. 16390897 10.1176/appi.ajp.163.1.109

[bib10] Flint J, Kendler KS. The genetics of major depression. Neuron 2014; 81: 484–503. 24507187 10.1016/j.neuron.2014.01.027 PMC3919201

[bib11] Cohen-Woods S, Craig IW, McGuffin P. The current state of play on the molecular genetics of depression. Psychol Med 2013; 43: 673–687. 22687339 10.1017/S0033291712001286

[bib12] CONVERGE consortium. Sparse whole-genome sequencing identifies two loci for major depressive disorder. Nature 2015; 523: 588–591. 26176920 10.1038/nature14659 PMC4522619

[bib13] Caspi A, Sugden K, Moffitt TE, Taylor A, Craig IW, Harrington H et al. Influence of life stress on depression: moderation by a polymorphism in the 5-HTT gene. Science 2003; 301: 386–389. 12869766 10.1126/science.1083968

[bib14] Kaufman J, Yang BZ, Douglas-Palumberi H, Grasso D, Lipschitz D, Houshyar S et al. Brain-derived neurotrophic factor-5-HTTLPR gene interactions and environmental modifiers of depression in children. Biol Psychiatry 2006; 59: 673–680. 16458264 10.1016/j.biopsych.2005.10.026

[bib15] Karg K, Burmeister M, Shedden K, Sen S. The serotonin transporter promoter variant (5-HTTLPR), stress, and depression meta-analysis revisited: evidence of genetic moderation. Arch Gen Psychiatry 2011; 68: 444–454. 21199959 10.1001/archgenpsychiatry.2010.189 PMC3740203

[bib16] Kim JM, Stewart R, Kim SW, Yang SJ, Shin IS, Kim YH et al. Interactions between life stressors and susceptibility genes (5-HTTLPR and BDNF) on depression in Korean elders. Biol Psychiatry 2007; 62: 423–428. 17482146 10.1016/j.biopsych.2006.11.020

[bib17] Baum AE, Solberg LC, Churchill GA, Ahmadiyeh N, Takahashi JS, Redei EE. Test- and behavior-specific genetic factors affect WKY hypoactivity in tests of emotionality. Behav Brain Res 2006; 169: 220–230. 16490266 10.1016/j.bbr.2006.01.007 PMC3762875

[bib18] Dugovic C, Solberg LC, Redei E, Van Reeth O, Turek FW. Sleep in the Wistar-Kyoto rat, a putative genetic animal model for depression. Neuroreport 2000; 11: 627–631. 10718326 10.1097/00001756-200002280-00038

[bib19] Pare WP, Redei E. Depressive behavior and stress ulcer in Wistar Kyoto rats. J Physiol Paris 1993; 87: 229–238. 8136789 10.1016/0928-4257(93)90010-q

[bib20] Pare WP. Open field, learned helplessness, conditioned defensive burying, and forced-swim tests in WKY rats. Physiol Behav 1994; 55: 433–439. 8190758 10.1016/0031-9384(94)90097-3

[bib21] Solberg LC, Baum AE, Ahmadiyeh N, Shimomura K, Li R, Turek FW et al. Sex- and lineage-specific inheritance of depression-like behavior in the rat. Mamm Genome 2004; 15: 648–662. 15457344 10.1007/s00335-004-2326-z PMC3764448

[bib22] Braw Y, Malkesman O, Dagan M, Bercovich A, Lavi-Avnon Y, Schroeder M et al. Anxiety-like behaviors in pre-pubertal rats of the Flinders Sensitive Line (FSL) and Wistar-Kyoto (WKY) animal models of depression. Behav Brain Res 2006; 167: 261–269. 16271773 10.1016/j.bbr.2005.09.013

[bib23] Malkesman O, Braw Y, Maayan R, Weizman A, Overstreet DH, Shabat-Simon M et al. Two different putative genetic animal models of childhood depression. Biol Psychiatry 2006; 59: 17–23. 16095569 10.1016/j.biopsych.2005.05.039

[bib24] Will CC, Aird F, Redei EE. Selectively bred Wistar-Kyoto rats: an animal model of depression and hyper-responsiveness to antidepressants. Mol Psychiatry 2003; 8: 925–932. 14593430 10.1038/sj.mp.4001345

[bib25] Mehta NS, Wang L, Redei EE. Sex differences in depressive, anxious behaviors and hippocampal transcript levels in a genetic rat model. Genes Brain Behav 2013; 12: 695–704. 23876038 10.1111/gbb.12063

[bib26] Williams KA, Mehta N, Wang L, Redei EE, Procissi D. Resting state functional MRI in a rat model of major depressive disorder. Neuroscience Meeting Planner: Society for Neuroscience, New Orleans, LA, Program No. 827.11. 2012.

[bib27] Hasler G, Northoff G. Discovering imaging endophenotypes for major depression. Mol Psychiatry 2011; 16: 604–619. 21602829 10.1038/mp.2011.23

[bib28] Andrus BM, Blizinsky K, Vedell PT, Dennis K, Shukla PK, Schaffer DJ et al. Gene expression patterns in the hippocampus and amygdala of endogenous depression and chronic stress models. Mol Psychiatry 2012; 17: 49–61. 21079605 10.1038/mp.2010.119 PMC3117129

[bib29] Pajer K, Andrus BM, Gardner W, Lourie A, Strange B, Campo J et al. Discovery of blood transcriptomic markers for depression in animal models and pilot validation in subjects with early-onset major depression. Transl Psychiatry 2012; 2: e101. 22832901 10.1038/tp.2012.26 PMC3337072

[bib30] Redei EE, Andrus BM, Kwasny MJ, Seok J, Cai X, Ho J et al. Blood transcriptomic biomarkers in adult primary care patients with major depressive disorder undergoing cognitive behavioral therapy. Transl Psychiatry 2014; 4: e442. 25226551 10.1038/tp.2014.66 PMC4198533

[bib31] Redei EE, Mehta NS. Blood transcriptomic markers for major depression: from animal models to clinical settings. Ann N Y Acad Sci 2015; 1344: 37–49. 25823952 10.1111/nyas.12748

[bib32] Fox C, Merali Z, Harrison C. Therapeutic and protective effect of environmental enrichment against psychogenic and neurogenic stress. Behav Brain Res 2006; 175: 1–8. 16970997 10.1016/j.bbr.2006.08.016

[bib33] Wright RL, Conrad CD. Enriched environment prevents chronic stress-induced spatial learning and memory deficits. Behav Brain Res 2008; 187: 41–47. 17904657 10.1016/j.bbr.2007.08.025 PMC2629380

[bib34] MacQueen G, Frodl T. The hippocampus in major depression: evidence for the convergence of the bench and bedside in psychiatric research? Mol Psychiatry 2011; 16: 252–264. 20661246 10.1038/mp.2010.80

[bib35] Kurtz TW, Montano M, Chan L, Kabra P. Molecular evidence of genetic-heterogeneity in Wistar-Kyoto rats - implications for research with the spontaneously hypertensive rat. Hypertension 1989; 13: 188–192. 2914738 10.1161/01.hyp.13.2.188

[bib36] Pare WP, Kluczynski J. Differences in the stress response of Wistar-Kyoto (WKY) rats from different vendors. Physiol Behav 1997; 62: 643–648. 9272677 10.1016/s0031-9384(97)00191-1

[bib37] Cancela LM, Rossi S, Molina VA. Effect of different restraint schedules on the immobility in the forced swim test: modulation by an opiate mechanism. Brain Res Bull 1991; 26: 671–675. 1933387 10.1016/0361-9230(91)90159-h

[bib38] Platt JE, Stone EA. Chronic restraint stress elicits a positive antidepressant response on the forced swim test. Eur J Pharmacol 1982; 82: 179–181. 6889973 10.1016/0014-2999(82)90508-8

[bib39] Tejani-Butt SM, Pare WP, Yang J. Effect of repeated novel stressors on depressive behavior and brain norepinephrine receptor system in Sprague-Dawley and Wistar Kyoto (WKY) rats. Brain Res 1994; 649: 27–35. 7953642 10.1016/0006-8993(94)91045-6

[bib40] Ayensu WK, Pucilowski O, Mason GA, Overstreet DH, Rezvani AH, Janowsky DS. Effects of chronic mild stress on serum complement activity, saccharin preference, and corticosterone levels in Flinders lines of rats. Physiol Behav 1995; 57: 165–169. 7878112 10.1016/0031-9384(94)00204-i

[bib41] Murray R, Boss-Williams KA, Weiss JM. Effects of chronic mild stress on rats selectively bred for behavior related to bipolar disorder and depression. Physiol Behav 2013; 119: 115–129. 23735843 10.1016/j.physbeh.2013.05.042

[bib42] Brenes JC, Fornaguera J. Effects of environmental enrichment and social isolation on sucrose consumption and preference: associations with depressive-like behavior and ventral striatum dopamine. Neurosci Lett 2008; 436: 278–282. 18400393 10.1016/j.neulet.2008.03.045

[bib43] Brenes JC, Fornaguera J. The effect of chronic fluoxetine on social isolation-induced changes on sucrose consumption, immobility behavior, and on serotonin and dopamine function in hippocampus and ventral striatum. Behav Brain Res 2009; 198: 199–205. 19027796 10.1016/j.bbr.2008.10.036

[bib44] Brenes Saenz JC, Villagra OR, Fornaguera Trias J. Factor analysis of Forced Swimming test, Sucrose Preference test and Open Field test on enriched, social and isolated reared rats. Behav Brain Res 2006; 169: 57–65. 16414129 10.1016/j.bbr.2005.12.001

[bib45] Green TA, Alibhai IN, Roybal CN, Winstanley CA, Theobald DE, Birnbaum SG et al. Environmental enrichment produces a behavioral phenotype mediated by low cyclic adenosine monophosphate response element binding (CREB) activity in the nucleus accumbens. Biol Psychiatry 2010; 67: 28–35. 19709647 10.1016/j.biopsych.2009.06.022 PMC2860655

[bib46] Richter SH, Zeuch B, Riva MA, Gass P, Vollmayr B. Environmental enrichment ameliorates depressive-like symptoms in young rats bred for learned helplessness. Behav Brain Res 2013; 252: 287–292. 23791932 10.1016/j.bbr.2013.06.021

[bib47] Crofton EJ, Zhang Y, Green TA. Inoculation stress hypothesis of environmental enrichment. Neurosci Biobehav Rev 2015; 49: 19–31. 25449533 10.1016/j.neubiorev.2014.11.017 PMC4305384

[bib48] Schloesser RJ, Lehmann M, Martinowich K, Manji HK, Herkenham M. Environmental enrichment requires adult neurogenesis to facilitate the recovery from psychosocial stress. Mol Psychiatry 2010; 15: 1152–1163. 20308988 10.1038/mp.2010.34 PMC2990187

[bib49] van Praag H, Kempermann G, Gage FH. Neural consequences of environmental enrichment. Nat Rev Neurosci 2000; 1: 191–198. 11257907 10.1038/35044558

[bib50] Teather LA, Magnusson JE, Chow CM, Wurtman RJ. Environmental conditions influence hippocampus-dependent behaviours and brain levels of amyloid precursor protein in rats. Eur J Neurosci 2002; 16: 2405–2415. 12492435 10.1046/j.1460-9568.2002.02416.x

[bib51] Nilsson M, Perfilieva E, Johansson U, Orwar O, Eriksson PS. Enriched environment increases neurogenesis in the adult rat dentate gyrus and improves spatial memory. J Neurobiol 1999; 39: 569–578. 10380078 10.1002/(sici)1097-4695(19990615)39:4<569::aid-neu10>3.0.co;2-f

[bib52] Leggio MG, Mandolesi L, Federico F, Spirito F, Ricci B, Gelfo F et al. Environmental enrichment promotes improved spatial abilities and enhanced dendritic growth in the rat. Behav Brain Res 2005; 163: 78–90. 15913801 10.1016/j.bbr.2005.04.009

[bib53] Furney SJ, Simmons A, Breen G, Pedroso I, Lunnon K, Proitsi P et al. Genome-wide association with MRI atrophy measures as a quantitative trait locus for Alzheimer's disease. Mol Psychiatry 2011; 16: 1130–1138. 21116278 10.1038/mp.2010.123 PMC5980656

[bib54] Biederer T, Sara Y, Mozhayeva M, Atasoy D, Liu X, Kavalali ET et al. SynCAM, a synaptic adhesion molecule that drives synapse assembly. Science 2002; 297: 1525–1531. 12202822 10.1126/science.1072356

[bib55] Zhiling Y, Fujita E, Tanabe Y, Yamagata T, Momoi T, Momoi MY. Mutations in the gene encoding CADM1 are associated with autism spectrum disorder. Biochem Biophys Res Commun 2008; 377: 926–929. 18957284 10.1016/j.bbrc.2008.10.107

[bib56] Takayanagi Y, Fujita E, Yu Z, Yamagata T, Momoi MY, Momoi T et al. Impairment of social and emotional behaviors in Cadm1-knockout mice. Biochem Biophys Res Commun 2010; 396: 703–708. 20450890 10.1016/j.bbrc.2010.04.165

[bib57] Costello DA, Lynch MA. Toll-like receptor 3 activation modulates hippocampal network excitability, via glial production of interferon-beta. Hippocampus 2013; 23: 696–707. 23554175 10.1002/hipo.22129

[bib58] Li X, Zhang W, Lencz T, Darvasi A, Alkelai A, Lerer B et al. Common variants of IRF3 conferring risk of schizophrenia. J Psychiatr Res 2015; 64: 67–73. 25843157 10.1016/j.jpsychires.2015.03.008

[bib59] Sequeira A, Klempan T, Canetti L, ffrench-Mullen J, Benkelfat C, Rouleau GA et al. Patterns of gene expression in the limbic system of suicides with and without major depression. Mol Psychiatry 2007; 12: 640–655. 17353912 10.1038/sj.mp.4001969

[bib60] Benyamin B, Middelberg RP, Lind PA, Valle AM, Gordon S, Nyholt DR et al. GWAS of butyrylcholinesterase activity identifies four novel loci, independent effects within BCHE and secondary associations with metabolic risk factors. Hum Mol Genet 2011; 20: 4504–4514. 21862451 10.1093/hmg/ddr375 PMC3196893

[bib61] Pinheiro EM, Xie Z, Norovich AL, Vidaki M, Tsai LH, Gertler FB. Lpd depletion reveals that SRF specifies radial versus tangential migration of pyramidal neurons. Nat Cell Biol 2011; 13: 989–995. 21785421 10.1038/ncb2292 PMC3149714

[bib62] Eisenberg D, Jucker M. The amyloid state of proteins in human diseases. Cell 2012; 148: 1188–1203. 22424229 10.1016/j.cell.2012.02.022 PMC3353745

[bib63] Ray B, Gaskins DL, Sajdyk TJ, Spence JP, Fitz SD, Shekhar A et al. Restraint stress and repeated corticotrophin-releasing factor receptor activation in the amygdala both increase amyloid-beta precursor protein and amyloid-beta peptide but have divergent effects on brain-derived neurotrophic factor and pre-synaptic proteins in the prefrontal cortex of rats. Neuroscience 2011; 184: 139–150. 21477639 10.1016/j.neuroscience.2011.03.067 PMC3391572

[bib64] Rosa ML, Guimaraes FS, de Oliveira RM, Padovan CM, Pearson RC, Del Bel EA. Restraint stress induces beta-amyloid precursor protein mRNA expression in the rat basolateral amygdala. Brain Res Bull 2005; 65: 69–75. 15680546 10.1016/j.brainresbull.2004.11.011

