# Supplementary material for: Nature and nurture: environmental influences on a genetic rat model of depression
Source: Transl Psychiatry. 2016 Mar 29;6(3):e770–. doi: 10.1038/tp.2016.28 (PMC4872452; doi:10.1038/tp.2016.28)
Supplement: Supplementary Information [file tp201628x1.pdf]

Supplementary Tables and Figures

Supplementary Figure 1. Frequency distribution of correlation coefficients of blood transcripts. Distribution of correlation coefficients in (a) WLI Control and WLI CRS and (b) WMI Control, WMI EE and WLI Control, WLI EE. The frequency distributions were obtained from correlation coefficients (r).

Supplementary Figure 2. Correlational expression networks. Unique co-expression patterns of blood transcript levels in (a) WLI CRS and (b) WMI EE animals. Two genes were considered to be co-expressed if their Spearman correlation was >0.7 and the p value < 0.05. Red lines represent positive correlations between two transcripts while blue lines represent negative correlations. Transcripts showing significantly different levels between (a) Control and CRS or (b) Control and EE by Bonferroni post-hoc are bolded and underlined.

Supplementary Table 1. Quantitative RT-PCR primer sequences
Supplementary Table 2. ANOVA results of chronic restraint stress
Supplementary Table 3. ANOVA results of environmental enrichment
Supplemental Table 4. Whole blood transcriptomic differences between WMI and WLI, Naive-CRS-Controls
Supplemental Table 5. Hippocampal transcriptomic differences between WMI and WLI, Naive-CRS-Controls
