# Supplementary material for: Nature and nurture: environmental influences on a genetic rat model of depression
Source: Transl Psychiatry. 2016 Mar 29;6(3):e770–. doi: 10.1038/tp.2016.28 (PMC4872452; doi:10.1038/tp.2016.28)
Supplement: Supplementary Table 1 [file tp201628x2.doc]

Supplementary Table 1. Quantitative RT-PCR primer sequences. F- Forward; R- Reverse

| **Gene** |  | **Sequence 5’ - 3’** | **Amplicon length (bp)** |
| --- | --- | --- | --- |
| *Adcy3* | *F* | GCG CAT AGG CAT GAA CAA AG | 57 |
|  | *R* | GGC TTC CGG GCT CCA AT |  |
| *Amfr* | *F* | GAT GAG AGA CAG CGC ATG TTA GTC | 73 |
|  | *R* | TGT TCA AGA ACC GCT TTC GA |  |
| *Atp11c* | *F* | TCT GAA GCT CGC CTT GGA TAC | 65 |
|  | *R* | GAG AAC CCC AAA TCA CAA AAT GA |  |
| *Cadm1* | *F*  *R* | TCC TGG TCC CTC CAC GTA AC | 70 |
|  |  | TCA ATC TCC TCC CCT TCA ACT G |  |
| *Cd59* | *F* | AAC AAA CAG CAC TTG CTC TCC TAA | 69 |
|  | *R* | TGA TAG ACT TGC TTT CCG GAT ACA |  |
| *Cdr2* | *F* | GGA GAG ACC AGC ACC AAG CT | 60 |
|  | *R* | AAG TGT TGG CGG AGG TCG TA |  |
| *Cmas* | *F* | CGG CTC GTT CTA CTT TGC TAA GA | 66 |
|  | *R* | GCC ATT TTC CCA CCC TGT AA |  |
| *Dgka* | *F* | GCA GTG TGG CCA AAA TAA TCA C | 61 |
|  | *R* | CTC ATG TCT GGC ACA CAG GTT T |  |
| *Fam46a* | *F* | CCA GAA ACC CCG AGG AAA TAA | 149 |
|  | *R* | GCT CTC CGA TGT CTG AGA AGT CA |  |
| *Gapdh* | *F* | CAA CTC CCT CAA GAT TGT CAG CAA | 118 |
|  | *R* | GGC ATG GAC TGT GGT CAT GA |  |
| *Irf3* | *F* | TGT GAT GGT CAA GGT TGT TCC T | 60 |
|  | *R* | CCT TCC CGG GCC ATC TC |  |
| *Kiaa1539* | *F* | CAC CAT CCA AGT GAC CTT ATT TAA CC | 71 |
|  | *R* | AGA AGT CAA AGG TCA CAA GGA ACA T |  |
| *Marcks* | *F* | TCT TTG TTG AAG AAG CCA GCA T | 131 |
|  | *R* | GCC ATT CTC CTG CCC ATT T |  |
| *Psme1* | *F* | GAG CTG ATG ACC AGC CTT CAT A | 65 |
|  | *R* | GAG AAG TAC TTA GAG ATC TGC GTT TGG |  |
| *Raph1* | *F* | CCT CTT TAT CCA GCT CCA GCA T | 67 |
|  | *R* | TTG GAC TGA GAC TCT GGG ATA CTG |  |
| *Tlr7* | *F* | CAT TCC TGA AAT GCC TCA ATT TG | 67 |
|  | *R* | TGG AGT TCA CTG CCA TTA AGA GTT |  |
