# Supplementary material for: Nature and nurture: environmental influences on a genetic rat model of depression
Source: Transl Psychiatry. 2016 Mar 29;6(3):e770–. doi: 10.1038/tp.2016.28 (PMC4872452; doi:10.1038/tp.2016.28)
Supplement: Supplementary Table 2 [file tp201628x3.doc]

| **Gene** | Blood | | | Hippocampus | | |
| --- | --- | --- | --- | --- | --- | --- |
|  | Strain | Treatment | Strain X Condition | Strain | Treatment | Strain X Condition |
| *Adcy3* |  |  | F = 5.5, p = 0.03 |  |  |  |
| *Amfr* |  |  |  |  | F = 12.62, p < 0.01 |  |
| *Atp11c* |  |  |  | F = 8.83, p < 0.01 | F = 34.91, p < 0.01 |  |
| *Cadm1* |  |  |  |  |  | F = 8.71, p < 0.01 |
| *Cd59* | F= 4.38, p < 0.05 |  |  | F = 37.39, p < 0.01 |  |  |
| *Cdr2* |  |  |  | F = 9.62, p < 0.01 |  |  |
| *Cmas* |  | 6.33, p < 0.05 |  | F = 10.00, p < 0.01 |  |  |
| *Dgka* |  |  |  | F = 4.52, p = 0.05 |  |  |
| *Fam46a* |  |  | F = 4.14, p = 0.05 |  |  |  |
| *Irf3* | F=10.49, p < 0.05 |  |  | F = 16.16, p <0.01 |  |  |
| *Kiaa1539* |  |  |  |  |  |  |
| *Marcks* |  |  |  |  |  |  |
| *Psme1* |  |  |  | F = 5.73, p = 0.02 |  |  |
| *Raph1* | F= 8.74,  p < 0.01 | F = 4.47, p < 0.05 | F = 7.60, p = 0.01 | F = 80.55, p < 0.01 | F = 15.78, p < 0.01 |  |
| *Tlr7* | F= 4.89, P < 0.05 |  | F =5.78, p = 0.02 |  |  |  |

Supplementary Table 2: ANOVA results of chronic restraint stress
