# Supplementary material for: Nature and nurture: environmental influences on a genetic rat model of depression
Source: Transl Psychiatry. 2016 Mar 29;6(3):e770–. doi: 10.1038/tp.2016.28 (PMC4872452; doi:10.1038/tp.2016.28)
Supplement: Supplementary Table 3 [file tp201628x4.doc]

| **Gene** | Blood | | | Hippocampus | | |
| --- | --- | --- | --- | --- | --- | --- |
|  | Strain | Treatment | Strain X Condition | Strain | Treatment | Strain X Condition |
| *Adcy3* |  |  |  |  | F = 26.32, p < 0.01 |  |
| *Amfr* | F = 5.23, p = 0.03 | F = 5.23, p < 0.01 |  |  |  |  |
| *Atp11c* |  |  |  |  |  |  |
| *Cadm1* | F = 7.91, p < 0.01 |  | F = 18.04, p < 0.01 |  | F = 6.64, p = 0.01 |  |
| *Cd59* |  |  |  |  |  |  |
| *Cdr2* |  | F = 6.87, p = 0.01 |  |  | F = 7.74, p < 0.01 |  |
| *Cmas* | F = 4.86, p = 0.04 |  |  |  |  |  |
| *Dgka* |  |  |  | F = 3.35, p = 0.03 | F = 5.35, p < 0.01 | F = 4.23, p < 0.05 |
| *Fam46a* |  |  |  | F = 5.91, p = 0.02 |  |  |
| *Irf3* |  | F = 10.64, p < 0.01 |  | F = 10.19, p < 0.01 | F = 10.19, p < 0.01 |  |
| *Kiaa1539* |  |  |  |  | F = 29.12, p < 0.01 |  |
| *Marcks* |  | F = 15.38, p < 0.01 |  |  |  |  |
| *Psme1* |  |  |  |  | F = 7.98, p < 0.01 |  |
| *Raph1* |  | F = 16.69, p < 0.01 |  |  | F = 10.93, p < 0.01 | F = 4.55, p = 0.04 |
| *Tlr7* |  |  | F = 4.78, p = 0.04 |  | F = 5.45, p = 0.03 |  |

Supplementary Table 3: ANOVA results of environmental enrichment
