# Supplementary material for: Nature and nurture: environmental influences on a genetic rat model of depression
Source: Transl Psychiatry. 2016 Mar 29;6(3):e770–. doi: 10.1038/tp.2016.28 (PMC4872452; doi:10.1038/tp.2016.28)
Supplement: Supplementary Table 4 [file tp201628x5.doc]

Supplemental Table 4. Whole blood transcriptomic differences between WMI and WLI, Naive-CRS-Controls

| **Gene** | **No FST Control Blood Transcript Levels**  ΔCt ± SEM normalized to WLI mean | | | **FST Control Blood Transcript Levels**  ΔCt ± SEM normalized to WLI mean | | |
| --- | --- | --- | --- | --- | --- | --- |
|  | WLI | WMI | p | WLI | WMI | p |
| ***Adcy3*** | **1.00±0.02** | **1.07±0.02** | **0.02** | **1.00±0.01** | **1.02±0.01** | **0.01** |
| *Amfr* | 1.00 ±0.07 | 1.02±0.06 | 0.82 | 1.00±0.03 | 0.96±0.05 | 0.48 |
| ***Atp11c*** | **1.00±0.01** | **0.91±0.02** | **0.00** | 1.00±0.01 | 0.99±0.01 | 0.62 |
| *Cadm1* | 1.00±0.02 | 0.97±0.02 | 0.21 | 1.00±0.01 | 0.97±0.02 | 0.22 |
| ***Cd59*** | **1.00±0.03** | **1.13±0.04** | **0.03** | **1.00±0.01** | **1.05±0.01** | **0.02** |
| ***Cdr2*** | **1.00±0.15** | **1.43±0.13** | **0.05** | 1.00±0.03 | 1.02±0.03 | 0.69 |
| *Cmas* | 1.00±0.03 | 0.94±0.04 | 0.25 | 1.00±0.01 | 0.97±0.01 | 0.10 |
| *Dgka* | 1.00±0.04 | 0.92±0.07 | 0.35 | 1.00±0.02 | 0.97±0.02 | 0.28 |
| ***Fam46a*** | **1.00±0.05** | **0.84±0.02** | **0.02** | **1.00±0.02** | **0.87±0.04** | **0.047** |
| *Irf3* | 1.00±0.05 | 0.91±0.06 | 0.26 | 1.00±0.03 | 0.92±0.04 | 0.08 |
| *Kiaa1539* | 1.00±0.06 | 0.95±0.02 | 0.46 | 1.00±0.03 | 1.02±0.03 | 0.62 |
| *Marcks* | 1.00±0.04 | 5.06±0.54 | 0.52 | 1.00±0.02 | 1.05±0.05 | 0.37 |
| *Psme1* | 1.00±0.01 | 0.98±0.06 | 0.99 | 1.00±0.02 | 1.01±0.02 | 0.64 |
| *Raph1* | 1.00±0.01 | 1.00±0.01 | 0.85 | 1.00±0.01 | 1.00±0.02 | 0.81 |
| *Tlr7* | 1.00±0.02 | 0.95±0.03 | 0.50 | 1.00±0.02 | 0.93±0.14 | 0.20 |

The delta CT values were normalized the to the WLI so that a qualitative comparison can be made between the two groups.

Bolded p-values indicate a significant difference between strains
