# Supplementary material for: Nature and nurture: environmental influences on a genetic rat model of depression
Source: Transl Psychiatry. 2016 Mar 29;6(3):e770–. doi: 10.1038/tp.2016.28 (PMC4872452; doi:10.1038/tp.2016.28)
Supplement: Supplementary Table 5 [file tp201628x6.doc]

Supplemental Table 5. Hippocampal transcriptomic differences between WMI and WLI, Naive-CRS-Controls

| **Gene** | **No FST Control Hippocampal Transcript Levels**  ΔCt ± SEM normalized to WLI mean | | | **FST Control Hippocampal Transcript Levels**  ΔCt ± SEM normalized to WLI mean | | |
| --- | --- | --- | --- | --- | --- | --- |
|  | WLI | WMI | p | WLI | WMI | p |
| *Adcy3* | 1.00±0.02 | 0.97±0.01 | 0.07 | 1.00±0.01 | 1.01±0.01 | 0.54 |
| *Amfr* | 1.00 ±0.03 | 1.17±0.08 | 0.09 | 1.00±0.02 | 1.05±0.02 | 0.11 |
| *Atp11c* | 1.00±0.02 | 1.02±0.01 | 0.29 | 1.00±0.02 | 0.99±0.02 | 0.65 |
| *Cadm1* | **1.00±0.01** | **1.10±0.03** | **0.004** | 1.00±0.02 | 1.01±0.03 | 0.74 |
| *Cd59* | **1.00±0.07** | **1.36±0.06** | **0.003** | 1.00±0.01 | 1.02±0.01 | 0.32 |
| *Cdr2* | **1.00±0.04** | **1.24±0.05** | **0.002** | 1.00±0.02 | 0.97±0.01 | 0.31 |
| *Cmas* | 1.00±0.02 | 1.03±0.01 | 0.28 | 1.00±0.02 | 0.99±0.02 | 0.66 |
| *Dgka* | 1.00±0.01 | 1.04±0.02 | 0.12 | **1.00±0.01** | **0.94±0.01** | **0.01** |
| *Fam46a* | 1.00±0.03 | 0.97±0.01 | 0.37 | **1.00±0.01** | **0.95±0.01** | **0.01** |
| ***Irf3*** | **1.00±0.03** | **1.28±0.11** | **0.03** | **1.00±0.02** | **0.91±0.02** | **0.01** |
| *Kiaa1539* | 1.00±0.00 | 1.03±0.01 | 0.06 | **1.00±0.01** | **0.97±0.01** | **0.04** |
| ***Marcks*** | **1.00±0.02** | **1.10±0.02** | **0.001** | **1.00±0.01** | **1.06±0.01** | **0.004** |
| *Psme1* | 1.00±0.02 | 1.03±0.01 | 0.26 | 1.00±0.02 | 0.98±0.02 | 0.36 |
| *Raph1* | **1.00±0.02** | **1.26±0.03** | **<0.001** | 1.00±0.01 | 0.97±0.02 | 0.26 |
| *Tlr7* | 1.00±0.02 | 1.04±0.04 | 0.45 | 1.00±0.02 | 1.03±0.02 | 0.23 |

The delta CT values were normalized the to the WLI so that a qualitative comparison can be made between the two groups.

Bolded p-values indicate a significant difference between strains
