# Supplementary figures and images for: Nature and nurture: environmental influences on a genetic rat model of depression
Source: Transl Psychiatry. 2016 Mar 29;6(3):e770–. doi: 10.1038/tp.2016.28 (PMC4872452; doi:10.1038/tp.2016.28)

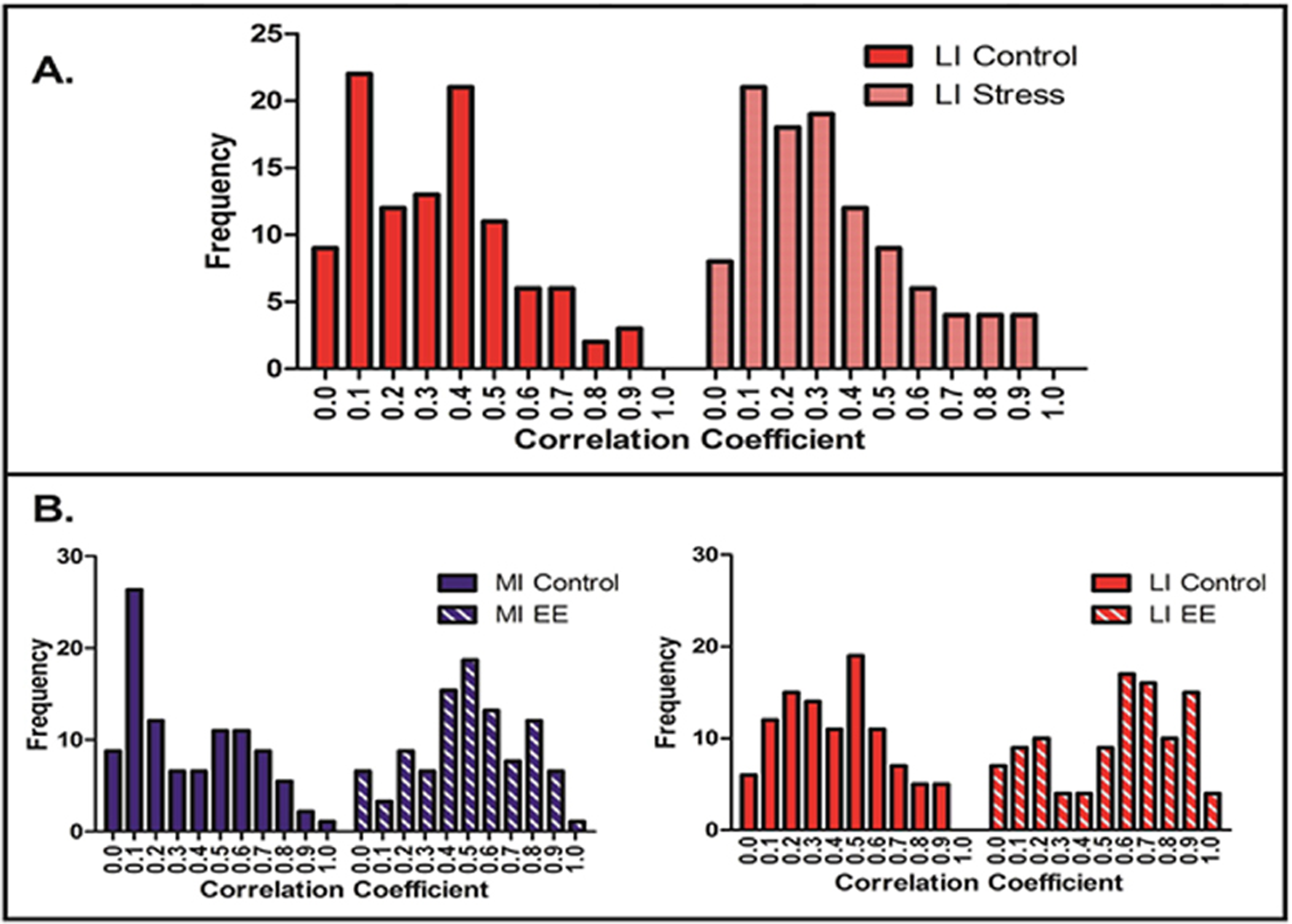

Supplement: Supplementary Figure 1 [file tp201628x7.tif]

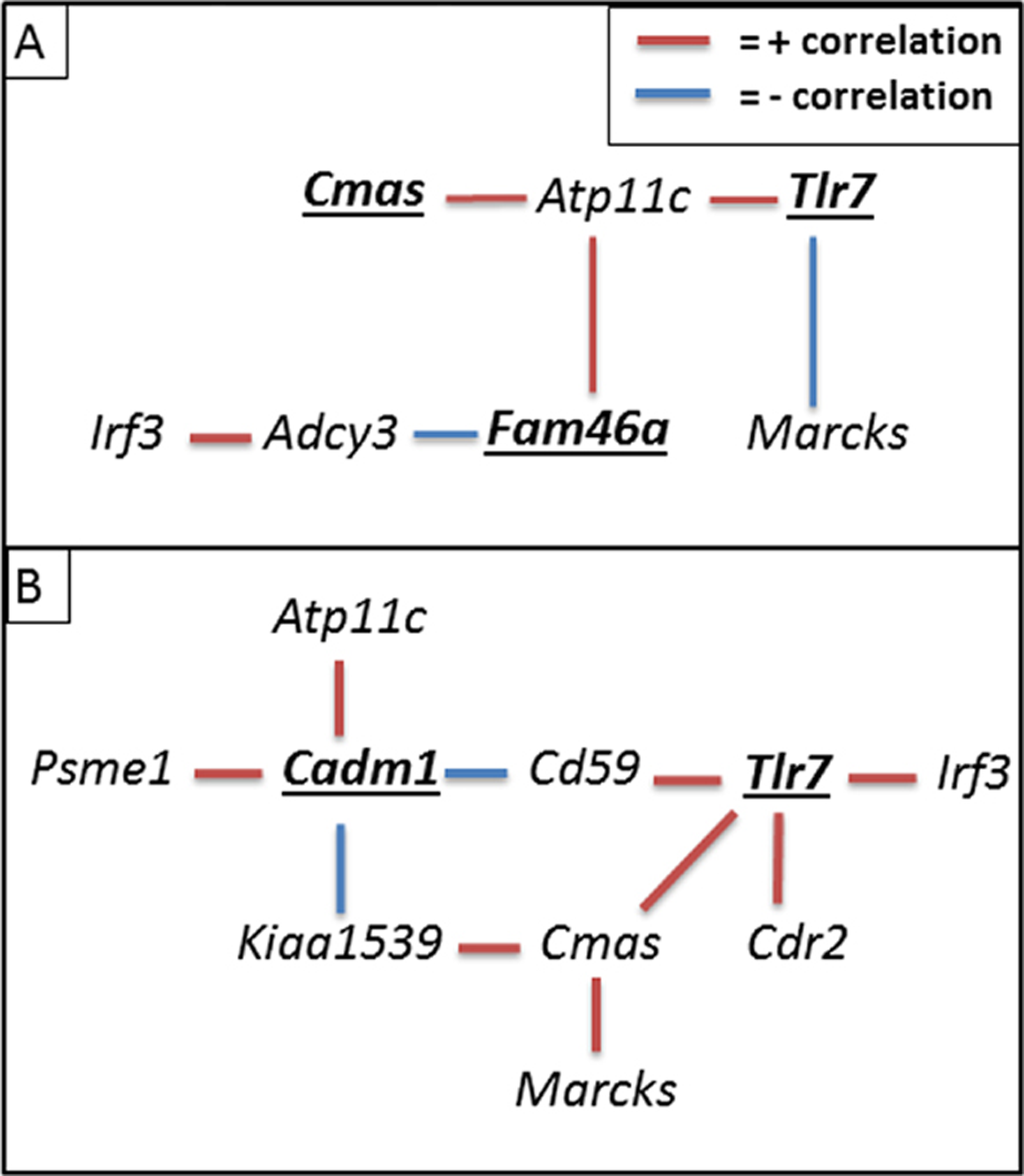

Supplement: Supplementary Figure 2 [file tp201628x8.tif]
